# Repellent Properties of δ-Octalactone Against the Tsetse Fly, *Glossina morsitans Morsitans*

**DOI:** 10.1673/031.008.4301

**Published:** 2008-05-27

**Authors:** Martin T. Mwangi, Nicholas K. Gikonyo, Isaiah O. Ndiege

**Affiliations:** ^1^ Department of Chemistry, University of Iowa. 423 CB, Iowa City, IA. 52241. USA; ^2^ Behavioural and Chemical Ecology Department. International Centre of Insect Physiology and Ecology. P.O. Box 30772-00506, Nairobi, Kenya; ^3^ Department of Chemistry, School of Pure and Applied Sciences, Kenyatta University. P.O. Box 43844, Nairobi 00100, Kenya

**Keywords:** allomone, bioassay, choice wind tunnel, olfaction, racemic δ-octalactone synthesis

## Abstract

δ-octalactone, produced by several Bovidae, has been suggested as a potential repellant of tsetse fly attack. Racemic δ-octalactone was synthesized via an abbreviated route. The product was assayed against 3-day old starved teneral female tsetse flies, Glossina morsitans morsitans Wiedemann (Diptera: Glossinidae), in a choice wind tunnel and found to be a potent tsetse repellent at doses ≥0.05 mg in 200 µl of paraffin oil (0.05 >p >0.01).

## Introduction

Avoidance of host-vector contact has been recommended as a method of choice for the control of vector borne diseases (WHO, 1996). This can only be achieved by use of chemical repellents and/or physical screens (nets, curtains, and clothing). Although the use of repellents (synthetic or natural) is appealing, their effect on the skin and the associated micro-fauna is a cause for concern and needs more careful and thorough studies. The amount of chemical repellents required for efficacy may be well above the toxic levels. Most organisms produce certain chemicals (allelochemicals) in minute quantities to ward off pests, vectors and parasites, as part of the natural defense mechanism system. Such chemicals are considered environmentally benign and may not interfere with the skin, micro-ecosystem or have toxic effects on the subject. Several efforts have culminated in identification of a few tsetse fly repellents from host animals, such as 2-methoxyphenol and lactic acid (Vale, 1980; Hargrove, 1991). Some were accidentally discovered from tsetse fly hosts while searching for attractants (Hassanali et al, 1986; Owaga et al., 1988; Bursell et al., 1988).

The tsetse fly shows differential host selection that is not dependent on their relative populations in an ecosystem (Weitz, 1963; Snow et al, 1988; Clausen et al., 1998). This phenomenon inspired the investigation of the semiochemical basis of the differential selection between ox, buffalo and waterbuck (Gikonyo et al., 2000, 2002, 2003). The feeding behavior of the tsetse fly, *Glossina morsitans morsitans* Wiedemann (Diptera: Glossinidae), on membranes treated with non-preferred (waterbuck) or preferred (ox and buffalo) hosts sebum indicated the presence of allomones in the former (Gikonyo et al., 2000). Investigation of the skin associated chemicals from the three animals led to identification of several EAG active compounds (Gikonyo et al., 2002, 2003). Semiochemical differences between the preferred and non-preferred hosts were established from GC-MS analysis of the sebum and trapped volatiles. The non-preferred hosts also contained the kairomonal aspects found in the preferred hosts except for a few EAG active compounds that differentiated them (Gikonyo et al., 2003). δ-Octalactone, guaiacol, 3-isopropyl-6-methylphenol and a series of (C_8_-C_13_) methylketones were found to differentiate the body surface chemistry of the waterbuck (non-preferred) from that of the ox and buffalo (preferred). From the volatiles of the waterbuck, δ-octalactone was characterized among other EAG active compounds (Gikonyo et al, 2002). Bioassay of a blend of all volatile components that differentiated the waterbuck from the ox and buffalo (δ-octalactone included) showed repellent properties against *G*. *m*. *morsitans* in a choice wind tunnel (Gikonyo et al. 2002). However, through the above mentioned studies, the repellent activity of δ-octalactone alone or its contribution to the repellent blend was not established.

**Figure 1.**
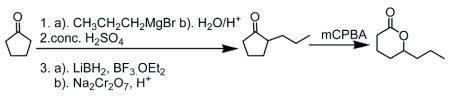
Synthetic scheme for of δ-octalactone

We hypothesized that δ-octalactone could be a potent tsetse repellent emitted by the waterbuck to ward off the pestiferous flies. Consequently, we developed an improved, high yield synthetic route to δ-octalactone. A simple achiral synthesis of δ-octalactone was developed and the lactone was assayed in a choice wind tunnel for repellency against 3-day-old teneral *G*. *m*. *morsitans*.

## Materials and Methods

### Synthesis of δ-octalactone

δ-octalactone was prepared from cyclopentanone according to the scheme in Figure 1. The reaction of cyclopentanone with *n*-propyl magnesium bromide followed by acid work up gave 1-propylcyclopentanol. Dehydration (*in situ*) gave *n*-propylcyclopentene. The olefin was oxidized to give 2-(*n*-propyl)cyclopentanone. Bayer-Villiger oxidation of the resulting ketone gave the target racemic δ-octalactone, (1.65 g, 11.62 mmol, 96.8% yield). MS *m*/*z* 142 (5%), 114 (10%), 99 (100%); 1H NMR (CDC1_3_) δ 0.9 (3H, t), 1.24 – 2.01 (8H, m), 2.39–2.58 (2H, m), 4.29 (1H, m); ^13^C NMR (CDCl3) δ 13.85 (C-8), 18.17 (C-7), 18.49 (C-3), 27.77 (C-2), 29.46 (C-4), 37.86 (C-6), 80.36 (C-5), 176.52 (C-1). Spectral data agreed with literature (Gikonyo et al, 2002)

### Insects

*G*. *m*. *morsitans* pupae were supplied by the International Livestock Research Institute (ILRI) in Nairobi and the International Atomic Energy Agency (IAEA) in Vienna and maintained in an insectary at 12 hrs light per day, 26± 1 °C, 70 ± 5% relative humidity until adult eclosion. Newly hatched flies were sorted out according to sex and experimental groups were starved while the rest were fed on pig blood through artificial membranes. The experimental group was transferred to a cylindrical cage and kept for three days before being used.

Behavioural assays were carried out in a cylindrical plexiglass choice wind tunnel, as previously detailed (Gikonyo et al., 2002, 2003). The wind speed in the tunnel was adjusted to 10 cm/sec, while the bioassay room was maintained at 26±1°C and 65±5% RH. The test odor consisted of the synthetic racemic δ-octalactone (2.5, 1, 0.5 and 0.1 mg) or an attractant (1 mg of a mixture of *p*-cresol, acetone and 1-octen-3-ol, in 4:100:1) in 200 µl of neat paraffin oil (Owaga et al. 1988;Paynter & Brady 1993; Gikonyo et al., 2003). A control containing 200 µl of neat paraffin oil dispensed within the alternate arm of the tunnel was included. Odor dispensers were made of clean black pieces of cloth tied on the open end of a plexiglass tube. The test odor in 200 µl paraffin oil was pipetted onto the cloth on one dispenser while the control dispenser was treated with 200 µl of paraffin oil. The wind tunnel was allowed to equilibrate for one minute, with all windows tightly closed and the fan on, before the test fly was introduced. Experiments were conducted in the late afternoon or early mornings to correspond with the natural host seeking rhythm. Behaviors monitored included: activation, initial direction of flight, distance covered during anemotaxis, walking and grooming.

**Table 1.**
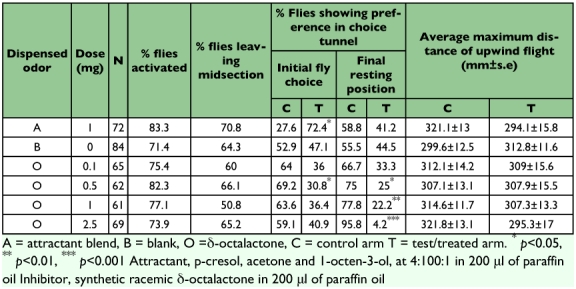
*Glossina morsitans* morsitans movement in a choice wind tunnel in the presence of δ-octalactone or attractant

Statistical analysis of differences in observed behaviors were performed non-parametrically using a χ^2^ test (SAS version 8e, 2000). Comparisons of proportions of tsetse fly distribution between the treated and the control arms of the wind tunnel were done using χ^2^. The percent of flies showing preference was calculated from those that were activated and not from the original number that were used in the experiment.

## Results and Discussion

Analysis of the initial direction of flight (Table 1) revealed no significant differences in fly distribution between the control and treated arms for blank, 0.1, 1.0, and 2.5 mg of δ-octalactone in 200 µl of paraffin oil. More flies initially opted for the control (69.23%, p< 0.05) arm at 0.5 mg of the δ-octalactone in 200 µl of paraffin oil. The initial direction of flight was dependent on the chemical dispensed (χ^2^ 26.1, p <0.05) suggesting that preference was influenced by the odor. However, the occurrence of a maximum in the number of flies avoiding the treated tunnel at 0.5 mg/200 µl suggests that there could be several factors influencing the choice of initial direction of flight. The effect of wind alone may induce positive anemotaxis by flies, but the presence of the δ-octalactone in the treated arm of the wind tunnel counteracted this effect. The observed trend indicates that the two factors have a maximum bias in favor of the repellent at 0.5 mg of the δ-octalactone in 200 µl of paraffin oil.

The final distribution of the flies on exposure to δ-octalactone (Table 1) showed dose dependency as indicated by the gradual increase in the proportion of flies showing preference for the control as the δ-octalactone dose increased. Significantly (*p*<0.05) more flies finally rested in the control arm when ≥0.5 mg of δ-octalactone in 200 µl of paraffin oil was dispensed. High amounts (1.0 and 2.5 mg) of dispensed δ-octalactone resulted in increase in the repellency. The final distribution of the flies was dependent on the amount dispensed (χ^2^ 20.054, p <0.05). This is consistent with the presence of an allomonal component in the choice wind tunnel (Gikonyo et al., 2002, 2003). For the attractant, the final distribution between the control and the treated was not significantly different (p >0.05). This may be explained by the absence of a host or an arrestant, prompting the flies to redistribute themselves randomly regardless of the odors emanating from both arms probably due to “frustration” in failing to get a host. At 0.1 mg of the δ-octalactone in 200 µl of paraffin oil, there were no significant differences (p >0.05) in initial direction of flight and final fly distribution suggesting that this dose has no significant repellency.

Parametric analysis of the maximum upwind distances covered by the flies using t-test (SAS version 8e, 2000) showed no significant differences between the control and treated arms irrespective of the odor dispensed (Table 1).

Analysis of these results show the potential repellent activity of the racemic δ-octalactone. The δ-octalactone was shown to be a tsetse fly repellent at ≥0.5 mg in 200 µl paraffin oil. However, significantly large amounts (1.0 or 2.5 mg per 200 µl) of δ-octalactone are required to make the flies stay away from the dispenser. This is consistent with earlier observations, when allomonal blends were dispensed in a choice wind tunnel bioassay and compared with kairomones and synthetic attractants (Gikonyo et al, 2002, 2003).

Although there can be varied differences between field and laboratory assays or between single components and blends, these data demonstrate that δ-octalactone elicit allomonal responses in the tsetse fly in a choice wind tunnel assay. Further work to confirm these results in the field is underway.
